# "The Hospital" Nursing Mirror

**Published:** 1900-06-02

**Authors:** 


### "The Hospital" Nursing Mirror.

Being the Nursing Section of "The Hospital."

[Contributions for this Section of "The Hospital" should be addressed to the Editor, The Hospital, 28 & 29, Southampton Street, Strand, London, W.C., and should have the word "Nursing" plainly written in left-hand top corner of the envelope.]

### Notes on News from the Nursing World.

### A FURTHER DISPATCH OF NURSES TO THE FRONT.

The Secretary of State for War informs us that the following nurses of the Army Nursing Service Reserve will embark for South Africa on Saturday in the mail steamer: Nurses A. Beith, A. P. Carruthers, J. E. G. Chapman, L. P. Dixon, M. S. Farley, M. E. Gillam, K. McGonigal, M. Potts, M. E. Scott, and E. S. Soley. Miss Lizzie Parrish Dixon was trained at the Royal Infirmary, Edinburgh, and has since been night nurse, and then assistant day nurse in the same institution. Miss E. Maude Halliwell received her training at the Royal Infirmary, Newcastle-on-Tyne, and afterwards joined the staff of the Northern Counties Nursing Home, of which home she remained a member until her appointment as sister to the Cork Military Hospital. Miss M. E. Scott was trained for three years at the Royal Albert Edward Infirmary, Wigan. She has since been charge nurse at the City Fever Hospital, Netherfield Road, Liverpool, and charge nurse at the Brook Hospital, Shooter's Hill, Kent. She joined the Army Nursing Service Reserve in December last, and was called up for duty at Netley in February. The fifteen nurses who were to have left in the "Lismore Castle" on Monday also leave in the mail steamer on Saturday. Miss A. F. Clarke, one of the fifteen, was trained at the Clinical Hospital, Manchester, and was afterwards five years staff nurse at University Hospital, London. She was ordered to the front in March, but was granted leave as she was nursing a dying brother.

### HEROISM OF NURSES AT MAFEKING.

Describing the incidents of the fighting at Mafeking, a correspondent of the *Daily News* says: "The work done by Miss Crawford, matron of the Children's Hospital, and Mrs. Buchan could not be surpassed for gallantry and devotion by anything in the war. Notwithstanding the bullets which our men were hailing upon the fort which the Boers had captured, Miss Crawford and her sister both left the Children's Hospital, which was about 300 yards away, and went to the assistance of the wounded." The peril which these ladies encountered was obvious, and their heroism is not the less great because it was unobtrusive. We also observe that in the first speech which General Baden-Powell delivered after the relief of Mafeking, thanking the inhabitants for the support they had given him, he complimented the nurses upon their pluck and devotion, shaking hands with Miss Hill, the matron of the hospital.

### ARMY NURSE MARTYRS.

No less than three deaths of war nurses have lately taken place in South Africa. Last week we announced that of Sister Florence Bell at Kimberley, and now we have to add to the obituary, with much regret, the names of Sister Stuart Jones and Sister Bonilla Hall, both of whom died from enteric fever at Bloemfontein. Miss Hall was trained at the Middlesex Hospital, and on September 29th, 1898, was appointed Sister of "Founder" Ward, which position she only vacated in March this year, in order to proceed to the front. Following so quickly on each other, these sad events can scarcely fail to provoke comment in the nursing world. It is true that nurses, no less than soldiers and surgeons, are exposed to danger and to death in the discharge of their duties, but we hope that these risks are, as far as possible, reduced to a minimum. In our issue for January 20th, Miss Wedgwood, the Matron of the Royal Free Hospital, in reply to a question whether she considered the nursing of typhoid cases to be attended by much risk, rejoined, "No nurse ought to get typhoid;" and she added, "Our regulations are so definite and so stringent, that if they are strictly adhered to there is substantially no danger." The fact that two nurses have died from the disease at Bloemfontein, however, shows that war nurses run war risks. The prevalence of typhoid fever among the troops has been due to conditions which have affected all who either accompanied or followed them, and, in addition to the risks run by the soldiers, we fear that at times it has been very difficult even in the hospitals for nurses to guard themselves against that "sick-room infection" from which in our English hospitals, as Miss Wedgwood says, no nurse ought to suffer.

### AN AMATEUR COME TO JUDGMENT.

The result of the visit of Lady Sykes, "in the smartest of nursing costumes," to South Africa, is to be found in the pages of a small volume in which she recites her experiences. She evidently did not get on well with the authorities, whether doctors or nurses, and this is, perhaps, the explanation of some of the extraordinary statements which the book contains, such, for example, as the following: "It was even frequently said in my presence," says Lady Sykes, "by many who were suffering or have suffered from snubs inflicted on their well-meant and charitable efforts, that army doctors, and particularly army nurses, would really almost imperil the lives of their patients rather than allow their wants to be relieved in an irregular and non-official manner." After this we are not surprised to learn, from the same high authority, that "the wounded soldiers were not only dependent upon the amateur nurses for their delicacies and comforts, but often for pure necessaries also." We suppose that this, and much more to the same effect, is intended as a sort of reply to the indictment of Mr. Treves. The average reader will more probably regard it as affording one of the most convincing proofs yet furnished of the incalculable mischief caused by the "Plague of Women" at the front. As a striking commentary on the experiences of Lady Sykes, we may refer our readers to the voluntary testimony of "A Grateful Nurse and

Mother" in our correspondence column, who alludes to the "splendid care and attention, night and day, shown by the sisters."

### THE NURSING QUESTION AT SCARBOROUGH.

THE Scarborough Board of Guardians have not yet succeeded in solving the problem of inducing their nurses to stop with them for any length of time. An attempt by the House Committee to carry a recommendation that probationers should be appointed for four weeks and then asked to enter into an agreement to stay for two years was defeated; and in place of it an amendment limiting the time to twelve months was carried by an overwhelming majority. One of the advocates of the amendment candidly confessed that he does not think the Scarborough Guardians are likely to secure the services of "the smartest and the best girls," and no doubt he is right. So long as assistant nurses in the Scarborough Workhouse Infirmary only receive a preliminary training, which does not fit them for higher posts, it is not at all probable that there will be a rush of "the smartest and the best girls" to that institution, notwithstanding the charms of Scarborough as a place of residence.

### QUEEN'S NURSES AT CHATHAM.

A FEATURE at the annual meeting of the Chatham Nursing Association was an address by Mrs. Goslette, lecturer to the Queen Victoria Jubilee Institute. She congratulated the Association on the fact that they command the services of the best trained nurses. She strongly advised that the schools in the district should be occasionally visited by them, as by that means disease was often detected in its early stages, and checked before it had time to develop. Dr. Holroyde, the medical officer, who was described by one of the speakers as "the guide, philosopher, and friend" to the Association, expressed his regret that skilful nursing is monopolised by the very rich and very poor, and his desire that some practical scheme could be found to introduce it into the homes of others. He suggested that it might be done "by some sort of provident fund, like the friendly societies." This opens up a question of great interest. The need of skilful nursing for persons who are neither very rich nor very poor does not, of course, require demonstration, but the difficulties in the way of providing it are at present considerable.

### NURSING AT THE TEMPERANCE HOSPITAL.

THERE have been several changes in the nursing staff at the Temperance Hospital recently. The opening of the ward last year necessitated an increase in numbers, and 36 nurses are now at work. Miss Wooll has recently been appointed night superintendent. She was trained at Guy's, and her last appoint- ment was at St. Saviour's Union Infirmary, Dulwich. Sister Barron has taken charge of the children's and male surgical wards, whilst Sister Richardson is now at Bloemfontein. She has been working at the Langman Hospital pending the arrival of No. 10 Hospital, of which she is temporary superintendent. Two other Temperance nurses are in South Africa—Nurse Pertwee, who is beyond the comforts of civilisation somewhere at Mooi River, and Nurse Davidson, who has lately arrived in charge of a transport, and who is returning to South Africa directly. With the augmented staff, the nursing home, so generously provided by Mr. Cadbury a couple of years ago, no longer affords enough bedrooms, and more are required. The nurses have one grievance. Whilst the training in the Temperance Hospital is acknowledged to be excellent, a well-known nursing society refuses nurses trained there a place in its ranks, on the ground that the hospital is a special, and not a general one.

### A NURSE AND HER NOTICE.

THE authorities of the Maidstone Union deducted a month's salary the other day in the case of a nurse who left the infirmary without notice. The excuse she urged was that "a very dear sister wished her to leave in order to reside with her," but this is not an adequate reason for throwing up an appointment without any regard to the conditions on which it was made, let alone the convenience of other people. In this instance the authorities were compelled to make a temporary engagement, and might have found it exceedingly awkward to provide for the requirements of the patients. We are always prepared to stand up for the rights of nurses, but it is their obvious duty to fulfil their obligations.

### EAST FINCHLEY CONVALESCENT HOME.

THE Convalescent Home at East Finchley, in connection with the Hospital for the Paralysed and Epileptic, has proved a great advantage to the parent institution. The patients derive great benefit from the bracing air; and Miss Headford, the matron of the home, has frequently the pleasure of welcoming members of the nursing staff of the hospital to recruit their health and strength. The nursing staffs of the home and hospital are quite separate, but the matrons are old friends. Miss Headford, who was trained at Canterbury Hospital, was night sister at the hospital from 1896 to 1897, and has held her present appointment for some months.

### IMPROVEMENTS AT THE GREAT NORTHERN CENTRAL HOSPITAL.

THE nurses and officers of the Great Northern Central Hospital have been very busy lately. Not only are all the beds full, but spring cleaning is only just approaching completion. The theatre sister is proud of the improvements in the operating theatre, which now presents a very spick and span appearance. The wooden seats, which were such an encumbrance, have been cleared away, the whole floor is now inlaid with terrazzio, and the electric light has been installed. The ward opened last year looks very well, but it is doubtful whether the round wards will become as popular with nurses as the oblong ones. The Great Northern Central was one of the hospitals which offered accommodation for the sick and wounded returning from the war, and the arrangements proposed were free from the objection that it was unfair to the poor to take beds provided for their use, since only the beds devoted to paying patients were offered to the soldiers. The nurses would have liked a share in tending the wounded heroes, but, as the home sister remarked, "I don't suppose we shall see them, they are so happily convalescent when they reach home." The full complement of nurses now all the wards are open is 11 sisters and about 30 probationers. The stabling provided for the bicycles of the staff is much appreciated.

### NURSING THE QUEEN'S TELEGRAPHIST.

"On the morning of the Queen's departure from Ireland," writes a nurse, "a patient was brought in on an ambulance to St. Mary's Hospital, who turned out to be the Queen's telegraphist. He had crossed over from Dublin to Holyhead the night before and came on to Euston by the morning train, in order to get back to Windsor Castle before her Majesty. Just as he was stepping out of a cab at Paddington Station he slipped, and a Pott's fracture was the result. We wondered what kind of a patient he would make, for, coming fresh from the glories and triumphs in Ireland, it did seem rather a rough drop to have to become just one of a crowd in a hospital ward. But there was nothing of the martyr about him. He was a veritable Job for patience and quite a host in himself, full of his visit to Ireland and the magnificent reception given to the Queen. Many were the inquiries from Windsor through the telephone as to his welfare. He went out last Friday in a beautiful khaki leather splint and on crutches."

### A TRIBUTE TO A QUEEN'S NURSE.

The Rev. Prebendary Digby Ram, vicar of Hampton, states that the nurse is the most popular institution in his parish, and receives the most cordial and general support. It is nearly three years since she was appointed, and her services have been so freely rendered alike to rich and poor, that Mr. Ram confesses he cannot think what Hampton would do without her.

### NO STARCH IN THEIR APRONS.

The Master of the Workhouse Infirmary at Bridgend is accused by Miss Lavinia Wilson of undue interference with the nurses. After only a fortnight's experience Miss Wilson resigned her appointment as nurse, a fact which is significant, because during the last few months several nurses have left the institution, and it has become exceedingly difficult for the Bridgend Guardians to secure the services of qualified nurses. In the letter announcing her resignation Nurse Wilson stated that it was not so much the place as the people in it which caused nurses to change so frequently. She went on to say that "the nurses are not allowed starch in their aprons," a deprivation which she attributed to the master of the workhouse. This, we presume, is but an example of the petty tyranny which prevails, and which no doubt explains the frequent changes in the nursing staff.

### A NOVEL EXPERIENCE BY A SCOTTISH MATERNITY NURSE.

Writing from a Scottish village a maternity nurse says: "I had rather a novel experience last week which I thought might interest some of the readers of the *Nursing Mirror*. A knock came to my door last Saturday, and when I opened it I was rather surprised to see a young woman of the gipsy tribe, who requested me to accompany her to a certain place about two miles outside of the village. On arriving at our destination, a beautiful spot in the corner of a meadow, I found the whole tribe encamped. I spoke a few minutes to the company, when I requested them to take me to the woman who was ill, and was politely told that this was my patient sitting on the grass at my feet. The woman then took me to their caravan, and a few hours afterwards she was safely delivered of a son. I attended her for three days, and on the fourth I found her up and dressed and sitting at the fire. After I had washed and dressed the baby I was told that my services would not be required again as they were leaving the district that night. But I was treated with the utmost respect and deference for the short time I was among them."

### THE SUNSHINE SOCIETY AT BELFAST.

Mention was made in the quarterly report of the Society for Providing Nurses for the Sick Poor, which was adopted at the meeting in Belfast the other day, of the work of the Sunshine Society. The report speaks of the cheering work done by the society among the convalescents and their friends, and says: "The first baskets were dispensed on the first Saturday of this month, and, we are sure, brought a large amount of sunshine and pleasure to many weary workers. The same kind friends who gave invitations to their own homes the past summers have renewed their invitations this year, when, no doubt, they will be equally appreciated. The bed at Bangor Home of Rest has again been engaged by the Sunshine Society, and is at present occupied by a patient whose back was badly strained by being thrown from an outside car. The doctor ordered her a change to Bangor before returning to work, so the Sunshine bed came in most opportunely." There is room for branches of the Sunshine Society on this side of the Channel.

### ONE OF MRS. LUDLOW'S PATIENTS.

Among the patients at present lying in the Royal Free Hospital, Gray's Inn Road, is Colour-Sergeant Roland Kensdale, of the 1st King's Royal Rifles. Kensdale arrived at South Africa just before the outbreak of the war, and was present at the battle of Dundee. He was fighting under Colonel Yule at Ladysmith on the day when the navy guns arrived, and there he was hit by a bullet in the left eye. "We had been on the march since nine o'clock at night, and at ten o'clock next morning I was lying down quietly shooting away when I suddenly felt as though my neighbour on the left had swung round the butt of his rifle catching me heavily in the eye; then I recollect crawling to a place of shelter, and then all was a complete blank until I found myself in bed in Ladysmith Town Hall being tenderly nursed by Mrs. Ludlow." Mrs. Ludlow—the wife of Major Ronald Ludlow, of the Army Service Corps—was the former matron of the Royal Free Hospital. Kensdale was one of three brothers taking part in the war, and all three were wounded, one of them losing a leg.

### SHORT ITEMS.

Miss Ada Giles informs us that the sale of "The Red Cross Nurse," which was mentioned in our columns, has realised £17 5s. for the War Fund, of which £12 12s. has been given to the Princess Christian's Fund. Mr. Walter Hedgcock set the verses to music, and they are published by Messrs. Cramer and Co.—The resignation by Miss Gardner of the post of matron at the Horton Infirmary, Banbury, is announced. Miss Gardner has held the position for five-and-twenty years. She was trained at the Nightingale School of Nursing, and has herself successfully trained a great number of probationers.

### Lectures on Medicine to Nurses.

By H. A. Latimer, M.D. (Dunelm), M.R.C.S. Eng., L.S.A.Lond.; Consulting Surgeon, Swansea Hospital; Past President of the South Wales and Monmouthshire Branch of Brit. Med. Assoc.; Past President of the Swansea Medical Society; Hon. Life Member of the St. John Ambulance Association, &c.

### STATE MEDICINE.

Continuing to speak on the subject of the general, as opposed to the particular, treatment of disease, I desire to draw your attention to that point in which the present generation is undoubtedly far in advance of those that have preceded it. Apart from any question of the drug treatment, in which I think there can be no opinion as to our present superiority over that of bygone days, the special features which distinguish medical practice now are those of preventative medicine, and the advent of the skilled nurse. These two factors have been of incalculable service to the community, and I propose to speak about them before passing to other parts of my subject.

The term preventative medicine speaks for itself, and carries its own definition. An alternative name for the same branch of the medical calling is "State Medicine," it being most justly held that it is entitled to this appellation as the work embraced by this department is performed for the benefit of the State at large, as opposed to the ordinary practice of the art of healing which applies itself to individual members of the same. Quite a specialism has arisen of late years in this work, and almost all the universities and qualifying bodies for registration as medical practitioners conduct examinations of medical men who are already qualified to practise their profession, on the special subjects of use in this branch of the calling, and grant degrees or diplomas marking the especial fitness of their possessors to take up public health work. I shall best give you an idea of the nature and scope of this work, which is carried on by the men who hold the appointments of Medical Officer of Health, by commenting on the characters of the examinations they are required to pass before they can obtain diplomas or degrees which are of extra service in enabling them to obtain those posts. In addition to their being already on the register as medical men, by which they have acquired the general education which qualifies them for that calling, they now specialize by studying the chemistry of air and water, so that they may be enabled by chemical and microscopical examinations to detect injurious bodies which may be present there; and particular attention is directed to bacteriology, which consists, as you know, of a knowledge of the micro-organisms which are now known to be so largely the cause of disease, especially of an epidemic character. As they have to know all about healthy dwellings, the theories respecting construction of houses, sanitary engineering, ventilation, water supply, and drainage have to be mastered; and as their duties entail advising sanitary authorities, they are required to have a knowledge of those laws which relate to public health. For the same reason they must show that they have studied the especial features which characterise epidemic and infectious diseases, and they must be trained in the effects produced by overcrowding in dwellings, vitiated air, and bad and unsuitable foods. Unhealthy occupations have to be noted, and they have to understand how nuisances can be abated; and, finally, statistical information as to the distribution of diseases within the United Kingdom, and the effects produced by various soils, seasons, and climates has to be mastered.

The result of this special education is more and more apparent as time passes, and as the places of insufficiently-educated men who held appointments at the creation of a sanitary service (and who are now dropping out of their posts by the efflux of time) are being replaced by new men of the higher education. I believe that we should obtain better results than we do even now if the law made it obligatory that all medical officers of health should possess special diplomas in sanitary science, and if they were paid properly and were debarred from the right of private practice as medical men. There ought, moreover, to be fixed tenure of appointments, for if there is one occupation more than another in which communities and private individuals are subjected to annoyance and expense at the hands of officials it is this one of public health. You cannot imagine how difficult it is to effect changes in dwellings and in properties generally where this has to be brought about by an expenditure of money. As I write this article I can call to mind several cases where I have lost attendances upon people by pointing out to the mean heads of families how necessary it was that the drainage of their houses should be put in sanitary order, as, by defects in the same, illnesses were being caused. The ordinary householder objects to be worried over such matters. If you can show him that his landlord will have to pay all expenses connected with the mending of a faulty sanitary service in his house he is content that you shall be an efficient adviser, but if he has to bear the cost of alterations and repairs he is apt to pooh-pooh your advice, and to tell you that you are over-officious and needlessly particular about drainage. And how much more truly does this apply to public sanitary service, especially to the holding of small appointments of Medical Officer of Health in little districts controlled by councils of small-minded men who are often holders of property coming under the review of the official in question. It requires no argument of mine to show that any person holding one of these posts should be irremovable from office—save from gross abuse of trust—and that he should be appointed to an area of sufficient size to allow of a salary being paid him which will secure his full services; when this is done, and the Medical Officer of Health is everywhere an independent person who can perform his duties without fear or favour, we shall have an ideal sanitary service.

Now let us see for a few minutes what conditions prevailed in England at the beginning of 1800, and contrast them with what obtains now; whilst doing so we can glance at some of the main sanitary enactments which have passed the Legislature, and which, by having become laws of the land, have effected results in general health of the happiest character. The earlier period I have spoken of coincided with marked changes in the incidence of population—changes which are still in progress and which are beginning to be a cause of much disquietude with thoughtful people. I allude particularly to the migration of population from country districts into towns. This movement led, at the beginning of the century, to a sudden and rapid increase in the size of provincial towns, and as a natural result, especially when it is remembered that few special laws then existed for the supervision of the health of communities, the dwellings became crowded in an unwholesome manner and the new ones which were erected were faultily constructed, by being built too near to each other, so that proper ventilation of them was well nigh impossible to be obtained, and the necessary drainage works were defective in the worst sense. Rows of houses were huddled together in the centres of towns in a manner which only those whose duties took them to these districts can have any conception of. Even now I know of courts in the town in which I live where rows of houses have high walls in front of them, separated only by about a distance of a yard and a half from the windows of the dwellings. Into such courts sun, light, and fresh air can only obtain entrance obliquely. Perched at one or both ends of the court will be the necessary closet arrangement, and this arrangement is for the common use of the inhabitants of the court.

### Nursing in the Civil Hospital, St. Helena.

By Miss Gertrude Wilson, Lady Superintendent.

When, five years ago, I made up my mind to apply for the post of lady superintendent at the Civil Hospital, St. Helena, I remember several people asking me, "What are you going to such a place for?" as if there could be no possible inducement to take such a post, either from a nursing or any other point of view. And yet we have been very happy here, have made many friends, and found abundance to do.

### The Nursing Staff.

As the original staff consisted only of myself and one other nurse, and I was to dispense for the hospital and the Government officials on the island, you will understand that we have seldom found time hang heavy on our hands. We were told that the average number of patients was six or seven, but we have accommodation for eighteen, and there are a great many days in the year when we have other than the average number, as, for instance, the last week, when we have had from thirteen to seventeen patients in, and many of them acute cases, there being an epidemic of pneumonia in the island at present. My staff nurse, who originally came with me, and who is fully trained and a qualified midwife, is still working here, but the Government has granted a third nurse, and we could often find work for more.

### The Building.

The hospital is an old building, three stories high. I am thankful to say we do not use the top storey for patients; stairs are a great increase of work. On the ground floor are a nice large dispensary, a little sitting room, two private wards, a good kitchen, and a ward boy's room. On the second floor are two nice, lofty, airy wards, one for men and one for women. Each holds eight beds, and has its own outside bath-room and lavatory.

Through the efficient help of H.E. Mr. Grey-Wilson, who was governor when we came, many improvements have been made. The kitchen has a first-rate range with a circulating boiler, and hot and cold water has been laid on upstairs. Lifts and speaking tubes have also been provided.

### The Nurses' Quarters.

The nurses' quarters consist of a cottage quite separate from the hospital, where we have bedrooms and a dining-room and kitchen. We keep our own servant, and have a monthly allowance instead of rations.

The patients contribute to the support of the hospital, and the Government makes up the deficiency. Sailors are paid for by the Board of Trade or their Consuls. The women and children of the troops are paid for by the War Office. The working people, many of whom belong to societies, pay 1s. per day. Those unable to pay are admitted on the parish or by means of tickets from a charitable fund called the Prendeville Fund, which has 650 tickets to dispose of in the year. The dispensary hours are from twelve to three for outsiders, and there is often a good deal to do, as we sell medicine at a very low rate to the poor, so that they often bring prescriptions to the hospital in preference to chemists.

### Night Work.

With regard to night work, I am afraid our arrangement is an unorthodox one. We take every third night on duty. We find that if one is put regularly on night duty the two left on day duty are so tied that it is difficult to get out for fresh air as one should. I do not think it is a good arrangement, but it is the best that we can manage. In slack times it is all very well, but when the work is heavy it is pretty hard. Several nights lately it has needed two to be up with delirious patients. Still, we keep our health fairly well on the whole, and nursing is not easy work anywhere. The climate is very healthy, though the summer in Jamestown is very trying. There is one great advantage to the place—namely, that by getting up into the hills for a few days you get a complete change of climate—it is so much cooler and damper. When the work is light, this is what we try to do by turns. Even two or three days in the lovely bracing air of the country picks one up.

### The Island not a Valley of Desolation.

I think people who only see the island from a steamer, or land only to pull up the steep, straggling, untidy street which forms Jamestown, go away with the idea that the place is a valley of desolation, surrounded by inaccessible rocks. But those who go up and inland have a different story to tell. Many parts are very fertile, green-wooded, picturesque in the extreme; lovely valleys running down to the sea; ridges of highlands bordered with pines and firs; cliffs with fern and bracken growing in their shelter; dells thick with arums and the large-leafed yam. Our wards are often lovely with flowers of many kinds brought mostly by old patients or their friends from inland. The visitor who said enthusiastically that St. Helena was an emerald set in granite was much nearer the truth than the average passenger who, judging from the deck of a steamer or the landing place, calls it a miserable hole.

### Lively Times.

Just lately we have been very lively in the shipping line. From two steamers a month, and an occasional man-of-war, we have become quite accustomed to seeing three or four steamers in the harbour at once. The "Powerful" put in here on her way home with the sick and wounded from Ladysmith. Transports with stores and cattle and defenders for the island, in the shape of the Gloucester Militia, arrive either from the Cape or England, and stay, perhaps, several days, giving us welcome opportunities of extra mails. Then we had the good fortune to have the hospital ship "Maine" here for two days, taking home wounded from Spionkop. Such a lovely ship, with every kind of appliance and such nice nursing sisters. I and my nurses have all been in Africa, and know the country well, so that we take a keen interest in the war.

### The Advent of the Boers.

The place is at present very much excited over the advent of the Boer prisoners, who are stationed in tents at Deadwood, near Longwood. Two transports have disembarked, and a third, with 1,100 Boers on board, lies in the harbour. There is a good deal of sickness among them, and there have been some deaths already. Those in good health, however, are profiting by the change and regular food. I do not fancy Mr. Steyn need worry about their quarters, which are comfortable and healthy. Every consideration is shown them.

### To Nurses.

We invite contributions from any of our readers, and shall be glad to pay for "Notes on News from the Nursing World," or for articles describing nursing experiences, or dealing with any nursing question from an original point of view. The minimum payment for contributions is 5s., but we welcome interesting contributions of a column, or a page, in length. It may be added that notices of entertainments, presentations, and deaths are not paid for, but, of course, we are always glad to receive them. All rejected manuscripts are returned in due course, and all payments for manuscripts used are made as early as possible at the beginning of each quarter.

### Bringing the Sufferers Home.

### A CHAT WITH ONE OF THE NURSES ON THE "LISMORE CASTLE."

By a Special Correspondent.

Underneath triumphal arches in honour of the relief of Mafeking, the flags and pennants trying not to look drenched by the day's rain, I journeyed to Kew, to hear something of the other side of war, the "seamy side," the side of suffering and death, from the nurse's point of view. And among groves of chestnuts and lilacs and laburnums, I found the house where Miss Schwekkard, one of the nurses who arrived here on the "Lismore Castle," was staying with her sister ere the ship again embarked for the Cape.

### The Voyage Home.

"Of course," she said, in answer to my inquiries as to the voyage, "you can't have everything on board exactly as you would in a hospital, but I must say that the arrangements on the 'Lismore Castle' were excellent. We had all we wanted, and a beautiful passage. Here is a photograph taken at Durban of my ward—'B. Ward' it was called—at least they say it is, but as we never wore strings to our Nightingale caps, I think that sister you see there must have been imported to look nice! It was so difficult to get washing done at Durban that we were as economical in the matter as possible. But I am sure these are my men in the cots."

"Had you not swinging cots? I see these are ordinary beds," I remarked.

"Yes, there were some; you see the ropes and pulleys at the further end; but the ward was fitted up for the occasion for fifty-six, and the beds were put in for the time; being fixed to the floor made them perfectly steady. And do you know, my patients never slept so well as one night when the sea was a little rough, I think the rocking motion soothed them. I noticed this particularly in the case of a poor boy to whom I had to give an injection every four hours; he slept beautifully that night."

### Heroes from Ladysmith.

"What they wanted most of all," Miss Schwekkard went on," was a good square meal; they were so hungry! I believe we must have brought the first detachment of men from Ladysmith, and the sufferings they had gone through there were too horrible. They were like skeletons when they came on board after living on horse-flesh and mealies. . . ."

"I suppose that by mealies you mean bread made from maize?"

"No, not even that; they had been eating just the maize itself, cooked, or made into porridge. Then on the way from Ladysmith to Durban, people all along the route, with every intention of being kind, had showered fruit upon them, bananas and red currants, and so forth—just the very worst things they could have had after enteric fever. Poor boys! It was pathetic to hear them. "Sister, do give me some plum-duff. I must have it even if I've got to die for it!" One poor boy was so anxious to get well and be put on ordinary diet that he tricked us over the clinical thermometer. It was left in his mouth, and when our backs were turned he took it out, so that when we returned it registered normal. He owned up to a chum of his afterwards, but it did him no good, for he was one of those who died. We had 10 deaths on board."

Miss Schwekkard went on to say that two of those who died were Roman Catholics, and that in their case one of the sergeants, also a Romanist, read the service; in the other cases it was read by the captain.

"But I didn't go to them all," she continued; "I simply couldn't bear it. It was so terrible to lose them, when we had done our very best to pull them through with champagne and chicken jelly, and every luxury we could think of. I know I was considered rather too soft-hearted, but I couldn't stand it; think what it would be if they had been one's own relations. And I am devoted to my patients, and have the greatest admiration for Tommy. He is so brave and generous to his comrades; in illness the soldiers will do anything for one another."

### The Ambulance Arrangements.

"Can you tell me anything about the ambulance arrangements from Ladysmith and Durban?"

"No, I saw nothing of the arrivals until they were on board, as we had to be in our places in the wards ready to receive them. But if there had been any possibility of nursing them without moving them so far it might have been the saving of some. I heard of one poor fellow who begged and implored to be taken out of the ambulance, the mere fact of being moved was such agony; but, of course, nothing could be done. Only a nurse knows what nurses have to go through in such cases as that. I wouldn't like anyone, however, to think that the bed-sores with which the patients arrived in England were due to the voyage; they were there when we received the men on board, and were particularly bad—of course, the result of what they had suffered in Ladysmith."

I had noticed in one of the papers the departure from the Cape of several men who were insane, and I asked Miss Schwekkard if she had seen anything of this, one of the most terrible effects of war. But the worst instance of this kind which she had known had been that of a man who suffered so intensely from depression, that it was not safe to leave him alone for fear he might attempt to jump overboard or injure himself in some way.

### The Silver Lining.

I am afraid Miss Schwekkard's South African experiences present a gloomy picture, but there is this "seamy side" in the midst of all the pomp and pageantry of war, and it is just this side that a nurse sees. Still, there was much in what I heard to brighten the picture—the devotion of the men to one another and to the sisters; their gratitude for all the care and attention bestowed on them by both nurses and doctors; and the joy of the home arrival of those who survive the horrors of war; these are the bright touches that relieve what would otherwise be too sombre.

### The Queen's Chocolate

"Will you put in a word for the Tommies?" Miss Schwekkard said when I was leaving. "Some of them got no chocolate; I don't know why, unless it was because they were shut up in Ladysmith. And those who had it do value it so. The boxes are most carefully treasured, or sent home to friends in England, and it seemed hard on my boys to have none. Can you say something about it?"

### Presentations.

Prescot Union Infirmary.—Miss S. J. Parrish, superintendent of nurses, who is leaving Prescot Union Infirmary to be married, has been presented with a silver toilet set (consisting of brush, comb, mirror, clothes-brush, and combined shoe-lift and button-hook) by the nursing staff, as a token of the esteem in which she has been held during the twelve months she has filled that position. Her departure will be much regretted by the nursing staff.

A Compliment to a District Nurse.—Miss Margaret Day, daughter of the late Rev. Maurice Day, vicar of Wickenford and head master of the Worcester Cathedral School, has, upon the occasion of her leaving Kidderminster, where she has worked as district nurse in the parish of St. Mary's for the last five years and six months, been made the recipient of a purse of gold and a handsome silver butter dish. Miss Margaret Day has done excellent work amongst the poor and sick.

### The Nurses of Plymouth Infirmary.

The following letter has been addressed to us, signed by seven members of the nursing staff of the Plymouth Infirmary:—

"We note that a paragraph in The Hospital of May 26th draws attention to nursing difficulties at Plymouth Workhouse Infirmary. At a meeting of the Plymouth Board of Guardians on May 22nd surprise was expressed at the number of resignations amongst the nursing staff, and some guardians professed ignorance as to the cause. Will you, sir, allow us to point out that the causes are well known, as will be seen by perusal of a letter which was sent to the board by the whole nursing staff on March 23rd, 1900, and to which no reply has been received. We also wish it to be understood that, provided we do our duty in a satisfactory manner, our medical officer and superintendent of nurses would be quite willing to give testimonials, and under such circumstances we think the board would do likewise. Our superintendent has always done her best to make us comfortable, but, as must be remembered, the infirmary is not separate from the workhouse, which increases the difficulty of administration."

The letter addressed to the Board on March 23rd is signed by the superintendent and nine other nurses. We append a copy:—

"Gentlemen,—We, the undersigned nurses, beg to request the attention of the Board of Guardians to the domestic and other arrangements affecting the nursing staff. We feel, and many of us have felt for a long time since, that the accommodation for nurses is not at all what we should expect, and is very much worse that that of most infirmaries. Our complaints are:—

"1st. That there is no sitting-room for nurses besides the dining-room, in which nine meals are served daily, and which consequently does not provide rest and quietness.

"2nd. That the kitchen is too small, and meals, therefore, unpunctual, and not so well served as they should be. Nurses get all the smell of cooking and conversation of the inmates.

"3rd. That the dormitories are not comfortable; none of them, except one, have a fireplace, and the partition walls (of match-wood) are so thin that there is no privacy. The noise from the bed-liers' ward is constantly heard, and there are most offensive smells from the emptying of slops from this ward on the landing of our bedrooms.

"4th. There is only one bath and one small w.c. for 11 nurses.

"5th. The nurse's bedroom opposite the L.-I. ward is extremely noisy day and night owing to loud conversation, &c., of the inmates.

"6th. We are exposed to draughts and wet feet in the cold and badly-drained yards between the blocks. One nurse has had a somewhat serious illness, and this we feel convinced is due to the exposure; other nurses often getting colds in consequence of frequent wetting.

"7th. Outdoor nurses have no accommodation in the house except for meals. No room for changing wet clothes and no privacy whatever. We feel we must ask the guardians to find us proper accommodation in the workhouse and not allow us to be exposed early morn and late at night to all weathers. We do not feel it right for us to have this lonely and wet exposure.

"8th. The rooms occupied by the superintendent are very noisy owing to close proximity of infirm wards.

"9th. Two of the outdoor nurses have made a complaint about sleeping arrangements, and this we hope will be altered without delay.

"We regret that we are forced to make a direct appeal to the board, as we do not see that any practical interest is taken in our surroundings, or that any steps are being taken to provide such accommodation for nurses as will enable them to have some privacy and rest when off duty."

### Wants and Workers.

A district nurse wishes to hire, or buy if possible, a spinal carriage, suitable for a boy 12 years of age.—Address Nurse, W.H.N.S., Winchmore Hill, N.

### A Nursing Sister's Visit to Ladysmith.

AFTER THE SIEGE.

Writing from Durban, an Army Nurse says: "The hospital ship 'Avoca' is having extensive alterations made; 150 hammocks are being removed and 84 beds put in their place, thus making another ward. Having a holiday I paid a visit to Ladysmith. I travelled up by the Princess Christian hospital train. The officer in charge most kindly pointed out places of interest. The line being a single one we progressed very slowly, and made many stops, which was good for sight-seeing. The two A.S.R. sisters were so nice to me that I greatly enjoyed the journey. At Ladysmith I visited Umbulwana Hill, where 'Long Tom' was mounted. The town looked gloomy, with damaged buildings and broken street lamps. My bedroom was the one used during the siege by Colonel Rhodes. It had three large holes, one in each of two walls and one in the floor. The Town Hall was wrecked; it had been used as a hospital. The saddest sight was Intombi Camp, with its 650 graves. The doctors and sisters felt it awfully that lives of enteric and dysentery patients were being lost during the siege for want of proper nourishment and stimulants. At one time they had only an average of a single tin of condensed milk to last twenty-four hours for thirty patients. After a most interesting time we returned here, and expect to leave with patients on board about June 1st."

### Appointments.

Whitworth Hospital, Darley Dale.—Miss S. Davidson has been appointed Nurse Matron. She was trained at the North Staffordshire Infirmary, Hartshill, Stoke-on-Trent, where, after three years' training, she was charge nurse for two years. Subsequently she was charge nurse at the Park Hospital, Hither Green; superintendent nurse at the Newcastle-under-Lyme Union Infirmary; and night sister at the Derbyshire Royal Infirmary, Derby.

Royal Bucks Hospital.—Miss M. Vaughan has been appointed Matron. She was trained at Wolverhampton General Hospital, of which she was afterwards sister. Subsequently, she has been charge nurse of the Verney Ward at the Royal Bucks Hospital.

Lindville Private Asylum, Blackrock, Cork.—Miss Thomson, formerly matron of James Murray's Royal Asylum, Perth, has been appointed Matron.

### Minor Appointments.

Isolation Hospital, Chester.—Miss Alice Hulme has been appointed Sister. She was trained at the General Infirmary, Chester, for three years, and was afterwards staff nurse. She has since been engaged in private nursing on her own account; charge nurse of the women's and children's wards in the Carnarvonshire and Anglesey Infirmary, Bangor; and assistant maternity nurse District Nursing Association, Llanelly, South Wales.

Isolation Hospital, Wimbledon.—Miss A. Streeter and Miss Kathleen Howard have been appointed Charge Nurses. Miss Streeter was trained at the Brompton Hospital for Consumption and St. Thomas's Hospital. She has since been staff nurse at Brompton and sister at East Dulwich Infirmary. Miss Howard was trained at the North Devon Infirmary, Barnstaple, and subsequently she has been charge nurse at Croydon Borough Hospital.

Poplar and Stepney Sick Asylum.—Miss Kate Florence Keeping has been appointed Night Sister. She was trained for three years at St. Saviour's Infirmary, East Dulwich, where she was afterwards staff nurse for a year. She has been ward sister at Poplar and Stepney Sick Asylum for two years.

### Echoes from the Outside World.

### AN OPEN LETTER TO A HOSPITAL NURSE.

HAD we not so recently exhausted our enthusiasm upon Mafeking Day and the Queen's Birthday I think we should have displayed more gladness over the information that Lord Roberts had crossed the Vaal. That at last our army should have invaded the enemy's country and have accomplished it with only four casualties, although the Boers are known to have assembled with 4,000 men at Englebrech Drift ready to oppose us, is news of much greater real moment than some over which we have been much more effusive in our plaudits. Commandant Eloff evidently thought we should find the feat more difficult than we did, for he assured a newspaper correspondent that our people "would not succeed in getting an entrance to the Transvaal in six months." The assurance that a British force was already over the river, which that correspondent must have been very pleased to give the bombastic young man, must have come as rather a blow. Happily, the mines at Johannesburg have been saved, even if it was intended to destroy them, by the arrival of Lord Roberts a day before he was expected. Many people think that the successful occupation of Johannesburg is the beginning of the end of the war; and, in any case, the British flag will now soon wave over Pretoria.

THESE are the days of big bazaars, but I think the National Bazaar has beaten the record, alike in its preparations, its attractions, the number of its visitors, and its receipts. Before it really came off there were folks who prophesied that the attendance the first few hours would not come up to expectation, because all but a very few rich people were too impoverished by the war to be willing to pay a golden sovereign for admittance to a show to which they were only asked so that they might spend freely. But the prophets were quite wrong, for during the period of the Princess of Wales's visit the crowd was simply enormous, and then alone it was estimated that no less than 3,500 visitors passed the turnstiles. The scene within was decidedly wonderfully effective, and must have been the result of much thought on the part of dozens of brains, and much labour on the part of hundreds of hands. It is impossible to describe adequately any of the sixty-six stalls, all housed under canvas, and all more or less resplendent, but the Flower Market especially was a dream. It was gay with bright draperies, supported by ivy-covered pillars, and lit by delicate pink electric-lighted shells, half-hidden in soft green festoons of smilax. The naval stall also particularly fascinated me. There were sailors on duty on either side, drapings of seaweed, decorations of shells and coral, ships in full sail and gaily painted withal, a piece of the wood from the old "Victory" for sale, and a "real Princess," like a fairy story, to preside over it all—Princess Louis of Battenberg.

MAFEKING, of course, played an important part, as it should in a war bazaar. "Mafeking Cup" figured among the drinks; the town itself had its place among the sweets fashioned out of marzipan, with marzipan Boers and marzipan shells in readiness to fire from marzipan guns, all in martial array, ready to attack the place, defended by the Town Guard, and watched over by General Baden-Powell looking through field-glasses, all manufactured from the same sweetmeat. Then the hero himself—on paper—sold well, 10s. being paid over and over again for an eighteenpenny photograph of the gallant defender, whilst Mrs. and Miss Baden-Powell did a roaring business because they were his nearest relatives. I did not see his mother, but am told she looks wonderfully young considering that she is a grandmother and has lately been through such awful anxiety, but his sister seemed to me to strongly resemble her brother's features we have learnt to know so well, though not in the flesh. The German Emperor's contributions, particularly the dozen engravings of his own pictures, each bearing an inscription "For the National Bazaar, 1900," in the Emperor's English handwriting, were much sought after. Mrs. Patrick Campbell, notwithstanding her recent widowhood, carried about kittens, recommending her wares as "Dear little kittens; only ten shillings, and worth ten pounds." As a matter of fact, some did realise as much as £8. But even this price was beaten by the £85 given for an American drink. The entertainments and the side shows were extensively patronised, the Princess of Wales attending the grand concert on the first day, at which Madame Albani sang and Mrs. Beerbohm Tree recited. She also witnessed a series of biograph reproductions. Her dress was, as it always is, in the best possible taste and thoroughly suitable for the occasion—a mauve and white foulard trimmed with mauve chiffon on the bodice and sleeves; a flower toque of mauve and yellow iris and Parma violets, with a touch of butter-coloured lace. A white chiffon boa added a soft, pretty touch to the costume.

TALKING of the Princess of Wales, the following little extract from a letter written in 1862, by an English girl at school in Germany to her friends in England, may interest you. The slanting, pointed caligraphy in the original is almost as old-fashioned as the phraseology. "They say here, more particularly our German governess, who has been nine years in Denmark, that it is wished that the Prince of Wales should marry a princess of Denmark. Our governess says that she is very beautiful, clever, and talented, and very amiable. Alexandra is her name, she is sixteen years old, and the daughter of the prince who will be King of Denmark when the present one dies. Our governess has her portraits, and she there looks very beautiful."

IN ordinary years the meet of the Coaching Club is a great feature in the fashionable world, and even if one is not a "Society" person the function is always worth going to, because the Park looks so lovely in its spring glory, and the horses are generally such beautiful specimens, and look so thoroughly aware of their position as the cynosure of all eyes. This year I did not intend to go, but as I had to get across the Park I made a slight detour so as to include the Magazine. I wished afterwards that I had not done so, for the departed glory of the show left me saddened and depressed. Instead of the gay, well-dressed throng who made movement along the paths almost impossible, there was only a scanty line of spectators, who looked neither in holiday trim nor in holiday spirits. The double row of carriages with laughing, chattering occupants was conspicuous by its absence; and instead of the thirty to forty coaches parading one after another, there were only sixteen which put in an appearance. Of course, the war was the principal cause of the lamentable falling off. One could almost feel the sorrow in the air. Even the drivers, who might in many cases have been proud of their teams, looked unusually grave, as if they, too, felt keenly the reduction in their ranks from absence and death. As someone wishing to make the best of things pointed out to me, perhaps the Grand Bazaar had also been a deterring element. Those who had attended at Kensington both days and were due again in the afternoon may have been too tired to care for anything but a quiet morning of rest at home.

### An Irish Soldier on the Nurses at the Front.

By a Sister.

At a time when the war and South Africa are in everybody's thoughts and hearts, a short journey I had with an Irish soldier may be of some slight interest, if only for the sake of Tommy's opinion and experiences of the Nursing Sisters with whom he came in contact.

I was off duty that afternoon, and was going up to London on private business in uniform. The afternoon was so dull, and the carriage so dark, as I entered the train two or three stations the London side of Woolwich, that it was not until we had started that I distinctly made out a limp figure wrapped in a dark cloak stretched full length upon the seat, with a khaki helmet drawn down over bent brows, and two crutches alongside. Then it scarcely needed the kindly explanation of a young gunner opposite that he was a wounded soldier from the front, who after being for five months in hospital—first at Pietermaritzburg, and since March 24th at the "Herbert" in Woolwich—was on his way home to Ireland. "He's not half strong yet," said the gunner, "and it seems a shame to send him along so far all by himself. I was not so lucky as he. I am on sick leave from India, where I was hurt playing cricket. I have two brothers though with French, and I daresay my turn will come next." Then, leaning forward, he raised the helmet from the other's eyes. "He had a drop of whisky before we started, and he can't stand it, he is still so weak, poor chap," turning again apologetically to his fellow-travellers. The poor young fellow opposite sat up and looked round. "Yes, I did have a little of that beastly stuff," and he made a wry face. "I was parting from some of my comrades—fine fellows, too, some of them—and I may never see them again. As a rule, I never touch whisky; it don't affect my head, but it has made me feel mortal queer here," touching his chest.

### One of the Inniskillings.

"A returned hero, are you?" asked a pleasant-faced man at the other end of the compartment. "Oh, I don't know about that," was the reply, as the curly-headed Irishman pulled himself together, and tried with manifest difficulty to put his feet down off the cushions, the pain of the effort being only too visible on the tired face; but he drew back his shoulders with decision. "I'm in the Inniskillings," which fact we had known before by the red lettering on his cap. "Where were you knocked over?" was the next question. "At Colenso, on December 15th. It was rather hard being down so soon. I was with White all along; but I can't grumble. I have two Boers to my account, and I hope to be back again soon, and do for plenty more." "You must go back for the siege of Pretoria," said another, "only don't let it be too long."

On being asked to tell us some of his experiences, he gave a graphic account of his share in the battle, how hotly the Inniskillings were engaged, feeling the shells even before they heard them; how he was hit by a ball, that entered his leg on the upper side, severing an artery, and passing through on the lower side, but he did not feel it; and how he was finally done for by a fragment of a shell. "Oh, it was bad to be lying out in the open, before they fetched us in, wondering whether we would be overlooked or forgotten. I emptied my pockets and gave eightpence to a Kaffir boy to look after me; but he just lay down by my side and went to sleep, the lazy beggar. But I have no reason to complain. They are a cowardly lot, those Boers; brave enough when their lines of communication are assured; but just sneaks! One aimed straight at my face—faith, but I just went at him with my bayonet, and gave him a good something, I know." "Did he recover from it?" "I don't know, and you can hardly expect me to say I care——" "What about the food—was it fairly decent?" "Oh, I can't growl in that way at all. You don't look for luxuries, but the want of water sometimes was terrible. I have known them dig for two hours before getting anything that was fit to drink. That was bad, I can tell you." Then he turned shyly to me. "Are not them flowers fine," he said, pointing to a bunch of lilac I was carrying; but he refused my offer of some, saying they had plenty of them "at home." Then, recognising my dress, he smiled a sunny smile that showed two even rows of white teeth. "Can't you sport the Red Cross?—you ought to have the Red Cross, Sister."

### The Nurses at the Front.

When some one asked whether he had suffered (as the papers had described it) from "a plague of women," he was quite indignant. "We want them all," he said. "I've been for five months in hospital, and the Sisters, they were good to me. Sister never forgot you. Sister S. and Sister R. were my special Sisters. It quite did you good to see Sister S. come smiling up to you. She was always smiling. Busy? Yes. And it was the night work that did for the ladies. So hot in the day that they could not sleep, and so awfully cold at night; but have a fire? not they, they wanted to be soldiers," with a bright laugh and glance at me. "I've read since I came home that Sister R. had died. Such a jolly, bright, go-ahead girl she was, and yet she died. I don't know why; she looked so well and rosy." Then he leaned forward again. "I'd rather have one of Sister's soft fingers than a thousand orderlies. Oh! they are well enough, but they go off to the canteen and forget you. Sister never forgets. It used to be a sight to see them come ploughing through the mud to see after the poor fellows." Did they wear white aprons? "They had green umbrellas," was the rather irrelevant reply.

### The Journey to Ireland.

How was he going to get to Ireland; "Oh, by the train, to be sure?" but such trivialities as to how to get to Euston were quite unconsidered. However, his pass was produced—Woolwich to Greenore—and the intermediate stages were evidently of no importance to the merry, happy-go-lucky Irish lad.

Of course, much discussion went on about the various events and mishaps that have occurred during these months of strife which it is needless to relate; then the carriage emptied gradually, and at Cannon Street, as an elderly gentleman got out he pressed the soldier's hand, saying, "Good-bye, and God bless you, my lad. Don't take any more of that whisky. I used to drink a good deal more of it than was good for me, but for the last two and a-half years have not touched a drop, and have never been so well in my life."

"I hope you won't think that I am a drunken soldier, sir. I'd be sorry for you to think that," and there was a suspicious shake in the man's voice.

When the train again started he limped across to me. "I am going to commandeer some of your flowers, Sister," and he broke off a spray of fresh lilac, which he fingered and smelt appreciatingly. "I used to steal Sister's flowers in hospital. I had the key of the door that led to her garden, and she used to wonder why I had always my bit of bloom fresh. I do think it a pity you are not a Red Cross; we want them all out there. Oh! it did us good to have Sister's kind face smiling at us," and a glance at his face showed how much suffering he must have gone through; indeed, from time to time his brows knit deeply at the twinges of pain.

A HELPING HAND.

When we reached Charing Cross I said, "You must let me help you a little if I can. I am a Sister, too, you know." So we got out and found his kit—three small canvas bags—and he began to hobble down the platform. "Stay with me for a few moments, will you, Sister?" he pleaded. I felt only too proud to do so. "How all these people are staring," he then remarked; "this is worse than all I have gone through. I hate being sent about like this. My leg does feel rotten; it feels double its own weight, but"—and again that sudden, cheery smile—"I'll be all right when I see my mother."

So we called a cab, and I turned to him: "I have just time to see you to Euston, if you like me to go with you." "Oh, I shall do very well"; but his tone encouraged me to add, "Of course, if you would rather I did not——" and I got a ready "I can't say I would not like it, Sister." So I got into the cab, prepared to help him, and taking charge of the crutches, my friend gripped the sides of the cab, but it was not so easy for him to step up as down, and an official came forward to assist him. "Can I lift you?" he said. "I can manage; thank God I have still two elbows, if only one good leg." Once he turned half apologetically to me. "You must have something else to do than to be looking after a chap like me." His one idea was to get back to the front—after having seen his mother—and to pay back to the enemy some of the old scores of treachery to his comrades in the past. At length Euston Station came in sight, and my slight connection with this brave soldier of the Queen was drawing to an end. We got out, and having my purse handy I paid the cabman, at which the Inniskilling demurred, until I said hastily, "You shall pay me back if you are too proud." Then he smiled, and said, "Well, Sister, I'll let you treat me, but I'd have liked to give cabby something." I put him in the care of a young porter, my leisure time being up and his train for Holyhead not being due for another couple of hours, and left him with another bit of lilac to remind him of home. His last words were, "Good-bye, and thank you, Sister; you ought to be a Red Cross. Anyhow, I've commandeered your flowers."

### The Nurses' Bookshelf.

[We invite Correspondence, Criticism, Enquiries, and Notes on Books likely to interest Women and Nurses. Address, Editor, THE HOSPITAL (Nurses' Book World), 28 & 29, Southampton Street, Strand, London, W.C.]

PRACTICAL NURSING. By ISLA STEWART, Matron of St. Bartholomew's Hospital, and HERBERT CUFF, M.D., F.R.C S., Medical Superintendent North London Fever Hospital, Tottenham. (William Blackwood and Sons. Price 3s. 6d.)

IT has been said that the nursing world is overcrowded with manuals on nursing, and that another at present would be quite superfluous. But this little volume certainly cannot be classed under that head, for it seems to meet a want created by the necessity of keeping nursing books up to date with the progress of nurse training. It is this want which has made so many formerly excellent guide books obsolete. Being a combined work of a practical matron and a medical superintendent, it deals with nursing from both points of view, and therefore is a distinct improvement on those handbooks written entirely by one or the other, and which often puzzle a probationer as to why in one handbook so much stress is laid on a point which the next handbook she reads entirely ignores. The arrangement of the book is excellent, a desirable point in preparing a book which is to be used as a text book by busy workers who require to obtain information on a point of nursing as speedily as possible, without having to wade through pages before alighting on what they seek.

In "Practical Nursing" the subjects are so well classified, and the headings all being printed in capitals, a busy probationer can readily turn up the chapter in which the subject is likely to be treated of, and in a few minutes find the particular paragraph she needs. The general character of the work is also a distinct advantage over so many nursing manuals, which seem to be written solely for nurses in a particular hospital, and lay down stringent rules for work which is done in this particular way only in the wards of that hospital. The book begins with an opening chapter by Miss Stewart, in which, in well chosen and practical words, much wise advice is given to probationers, advice which it would be well for them to carefully follow, and which, if taken to heart, will save them much future disappointment. The spirit of it recalls the wise teaching of Miss Florence Nightingale, and shows in what school and under what influences Miss Stewart was trained. The rest of the book is divided into 16 chapters, treating of some 13 different nursing subjects. Some of these chapters are so thoroughly practical and contain so much important information that it would be well if nurses learnt them by heart. Notably Chapter II., on "Ward Hygiene," also Chapter XVI., on the "Production of Surgical Cleanliness," though it seems a pity that in this so little stress is laid on the necessity of personal surgical cleanliness of the nurses attending at an operation. If the second volume is as good as the first, "Practical Nursing" will be a most useful handbook for nurses, and should have a successful career.

THE PRIVATE NURSE: SOME REMINISCENCES OF EIGHT YEARS' PRIVATE NURSING. By JESSIE HOLMES. (Published by Mr. T. Fisher Unwin, 11, Paternoster Buildings, London, E.C. Price 3s. 6d.)

MISS HOLMES's brightly-written reminiscences give a pleasant insight into the varied scenes which go to make a nurse's life. Incidentally, the writer takes her readers into her confidence, and shows them glimpses of her girlhood's dream to become a hospital nurse; how in those early days she thought only of the "poetry of nursing," which in her mind "consisted of shaking up pillows, reading delightful stories to interesting patients, wearing a becoming uniform and a jingling chatêlaine." She found the poetry, it is true, but intermingled with so much prose that she is "firmly convinced that unless a woman is prepared to enter heart and soul upon her work she ought not to become a nurse." She herself was of the stuff real nurses are made, for she seems to have won her patients' good-will, and to have nursed with equal pleasure and success the rich and the poor, schoolboys and professors, chronic invalids and undergraduates, dipsomaniacs, typhoid cases, and old age. The "cases" are lightly and humorously sketched, and medical details are agreeably conspicuous by their absence. During eight years' hard work Miss Holmes only broke down twice. Her general good health she attributes to her practice of taking a thorough holiday every year. How keen was her enjoyment of nature may be gleaned from the final chapter of her book, wherein with a light hand she sketches many an amusing incident of her travels. Perhaps of the most laughable is one used to illustrate the quickness of the donkey boys of Cairo. The donkeys enjoy a succession of remarkable appellations, often chosen on the spur of the moment in order to recommend a steed to a possible rider. Thus the donkey boys, seeing that Miss Holmes was a nurse, waylaid her with earnest entreaties to have a ride upon "Florence Nightingale." Upon her refusal one of the rejected animals was subsequently hired to another lady as "Ta-ra-ra-boom-de-ay." Nurses will be well amused by the prettily set forth little volume; we only fear lest intending nurses may gather from its pages an exaggerated idea of the "brightness" of the life before them.

### Everybody's Opinion.

[Correspondence on all subjects is invited, but we cannot in any way be responsible for the opinions expressed by our correspondents. No communication can be entertained if the name and address of the correspondent is not given, as a guarantee of good faith but not necessarily for publication, or unless one side of the paper only is written on.]

### THE RELIEF OF MAFEKING—AT A FEVER HOSPITAL.

The Medical Superintendent of the Grove Hospital, Lower Tooting, writes: I desire to correct a slight inaccuracy in your account of the rejoicings at the Grove Hospital over the news of the relief of Mafeking. Three of the porters, after fixing the flagstaff on top of the Water Tower, sang "God Save the Queen," but none of the nurses or female staff were with them, and no dancing took place there at all. I feel bound to ask you to make this correction as the account might otherwise create a false impression of the behaviour of the staff inside the hospital.

### AMATEURS AND ARMY NURSES.

"A Grateful Nurse and Mother" writes: Having an only son at the front who has been ill with enteric, I wish it to be generally known how grateful he is for the kind, considerate attention he received night and day from the nurses during his stay at Grey College Hospital, Bloemfontein. On a former occasion he had enteric after Tirah, and was nursed in his own home in India by his wife and other relations; but, though he had all the love and care possible, the attention he received at Bloemfontein far exceeded all they did for him. A friend went to see him in Grey College Hospital, and writing to me since, says, "How very fortunate —— was to be ill where he could receive such splendid care and attention night and day; nothing could be better." How grateful I am words fail me to tell; but, hearing and reading so much about the things left undone, I thought that a little about what is done would be acceptable.

### THE USE OF THE OBSTETRIC BINDER.

Nurse Enid writes: The diversity of opinion as to the efficacy of the binder in monthly nursing seems to be of recent growth. At one time the patient was bound as a matter of course, and in my opinion, after some years as a maternity nurse, all patients should wear a binder for at least a fortnight after delivery. After that time the parts should be in their proper position, and if the patient has made a good recovery the binder can then be safely left off. I like my patients to wear Thorne's Salmon Binder. I consider it by far the best. Of course it can only be worn for the fortnight the patient is in bed; at the end of that time I take it off and put on a thin binder, which the patient wears for a few days as she moves about the house. This is then taken off, and I have never known evil consequences to follow. The secondary hæmorrhage of which "Monthly Nurse" speaks may arise from several causes, and the use or disuse of the binder have nothing to do with it. For instance, if the case were a forceps one, it is just possible that a small piece of the membrane lining the uterus might be torn, and apparently heal with the rest of the parts, but when the patient stands up that small place bursts, and so brings on hæmorrhage. But these cases are not very common, so great is the care used by doctors, and "Monthly Nurse" need not worry unnecessarily; she has been unfortunate in having three cases so near together. The latest medical idea is, that, given a tractable patient who will lie quite still and do as she is told, the parts will resume their proper position without a binder. But how many gentle tractable patients do we get? Not one in ten. Finally, I do not consider that the figure of the average Englishwoman is suited to the binderless condition. As time goes on she "spreads" below the waist. Can anything be uglier than the pendulous abdomen in a woman of middle age? And are we going to produce this feature in our young women owing to this latest fad—for it is nothing else—of doing without the binder? Most of us know that after the second child there is a tendency to be pendulous. A Jersey lady was much amused at our habit of binding. She told me she had ten children, and had never worn a binder. I expressed my surprise that her figure was so good, when she admitted that it was customary in the Channel Islands to wear a long tightly-laced corset. I prefer the binder, and I do not in ordinary circumstances think it necessary to ask the doctor's permission to discard it.

### Trooping the Colour.

"I got off duty," writes a nurse, "with a rush at ten o'clock, flew to my room and changed, for I had a balcony ticket to see the trooping of the colour. It was delightful to feel smart again in a new frock, so I indulged in a hansom. After showing my treasured ticket to the huge Guardsman at Whitehall gate, and wondering to whom I was to show it next, I was taken gently by the elbow, and an officer in the brilliant uniform of the Queen's Household said, 'Just wait half a minute, because the King of Sweden is in this carriage; he is going to get out.' Instantly some soldiers had formed a double line from the carriage door to an entrance in the wall, and I was standing inside by a burly policeman. The officer went forward, shook hands, and conducted the tall, heavy, tired-looking gentleman up the stairs. The line melted and let in the jostling, well-dressed crowd, and I could hardly believe that it was a real, live king who had passed. Two minutes later I stepped out of a trap-door on to the leads of the north balcony. The Parade, in its gleaming glory, with the sun glittering on the bayonets, was before me, and I forgot for a while everything but that this was my heritage, and I was a soldier's daughter. Then the band played, and we heard a distant roar, which told us that the staff were nearing. They came over the parade-ground nearer and nearer, till we could distinguish each individual. We heard all round: 'There is the Prince of Wales, with the dear old Duke.' 'Is that the Duke of York, in a helmet?' 'Who is the gentleman in khaki?' 'Lord Wolseley looks delightful.' But there was a great heart-ache in their voices. No one mentioned them, but everyone thought of our brothers in South Africa, and some of our sisters, too. The parade was so pretty, the trooping so simple. The men looked firm and strong, the officers trim and neat. The band was beautiful, but played ballads this year instead of the old march pasts. There was no appplause; everyone watched in silence instead, and rose when 'God Save the Queen' was played. Then the Prince came forward and saluted with his sword. It was over, and the rain came. The people turned for shelter, but the troops stood calmly waiting, while the Staff rode away, and one by one the Royal carriages rolled across the parade-ground. As for me, I slipped through the crowd and arrived at the hospital just in time to go on duty at noon."

### Where to Go.

Exhibition of the Royal Photographic Society, 66, Russell Square, ten to four; Wednesday, ten to eight.

### TRAVEL NOTES AND QUERIES.

Rules in Regard to Correspondence for this Section.—All questioners must use a pseudonym for publication, but the communication must also bear the writer's own name and address as well, which will be regarded as confidential. All such communications to be addressed "Travel Editor, 'Nursing Mirror,' 28, Southampton Street, Strand." No charge will be made for inserting and answering questions in the inquiry column, and all will be answered in rotation as space permits. If an answer by letter is required, a stamped and addressed envelope must be enclosed, together with 2s. 6d., which fee will be devoted to the objects of the "Hospital Convalescent Fund." Any inquiries reaching the office after Monday cannot be answered in "The Mirror" of the current week.

Scilly Islands (L.W.)—Thanks for your kind and appreciative letter. So far the Scilly Isles are not suitable for those of slender means. Accommodation (no competition) is not cheap, and there is considerable difficulty in reaching or leaving St. Mary's in rough weather. In summer a boat goes three times a week from Penzance (weather permitting), fares 7s. and 5s. I rather think you would be disappointed, the Islands are so small that one feels cramped. Armorel's position and that of an occasional tourist are very different you know. It might be worth while to spend a week on the Islands if you had a month in Cornwall, but it would be expensive, but not to spend the whole of your holiday there.

### For Reading to the Sick.

### WHITSUNTIDE.

"IF I go not away the Comforter will not come unto you; but if I depart I will send Him unto you."—*St. John* xvi. 7.

Come, Thou Holy Spirit, come;
And from Thy celestial home
Shed a ray of light Divine;
Come, Thou Father of the poor,
Come, Thou source of all our store,
Come, within our bosoms shine:

Thou of Comforters the best,
Thou the soul's most welcome guest,
Sweet refreshment here below;
In our labour rest most sweet,
Grateful coolness in the heat,
Solace in the midst of woe.

O most Blessèd Light Divine,
Shine within these hearts of Thine,
And our inmost beings fill;
Where Thou art not, man hath nought,
Nothing good in deed or thought,
Nothing free from taint of ill.

Heal our wounds; our strength renew;
On our dryness pour Thy dew;
Wash the stains of guilt away:
Bend the stubborn heart and will,
Melt the frozen, warm the chill;
Guide the steps that go astray.

—*From the Latin.*

**Reading.**

The Spirit coming forth at Pentecost out of our Lord's uplifted manhood, as from a glorious fountain of new life, perpetuates all its richness, its power, its fulness in the organised society which He prepared and built for the Spirit's habitation. The Church, His Spirit-bearing body, comes forth into the world, not as the exclusive sphere of the Spirit's operations, for "that breath bloweth where it listeth," but as the special and covenanted sphere of His regular and uniform operation, the place where He is pledged to dwell and to work; the centre marked out and hedged in, whence ever and again proceeds forth anew the work of human recovery; the home where, in spite of sin and imperfection, is ever kept alive the picture of what the Christian life is, that is, of what common human life is meant to be and can become.—*Canon Gore.*

If we look higher still, we do indeed behold our Lord setting an example; but we observe also that there is something which He appraises higher than this function of example. Had this been His highest work, it would, beyond a doubt, have been expedient for us, if possible, that He should not have gone away. As it was, it was "expedient" that His disciples should lose His visible example, that they might gain a greater gift—the gift of the Spirit. "If I go not away, the Paraclete will not come unto you; but if I go I will send Him unto you." In fact, the Paraclete did come at Pentecost, and in virtue of His coming the Church became a body instinct with a new life, and Christianity a thing "not in word, but in power."—*Ibid.*

All that has been felt from the first springing of our regenerate life, the kindlings of hope, the visions, the resolve, the sense of power, the openings of heaven, the intimation of the will of God, growing with our growth, and strengthening themselves in meditative hours, in sweet communion with God . . . ever growing prospects of advancing grace, as we gain firmer hold of the unseen world—these are all the results of the Revelation of the Blessed Spirit.—*Rev. T. T. Carter.*

Grant us Thy peace throughout our earthly life,
Our balm in sorrow, and our stay in strife;
Then, when Thy voice shall bid our conflict cease,
Call us, O Lord, to Thine eternal peace. —*Ellerton.*

### Notes and Queries.

THE Editor is always willing to answer in this column, without any fee, all reasonable questions, as soon as possible.

But the following rules must be carefully observed:—

1. Every communication must be accompanied by the name and address of the writer.
2. The question must always bear upon nursing, directly or indirectly.

If an answer is required by letter a fee of half-a-crown must be enclosed with the note containing the inquiry.

*Maternity.*

(82) Will you kindly tell me if a nurse holding a certificate for maternity training, but not for general nursing, though with four or five years' experience in a nursing home, would find it difficult to obtain a post as district nurse? 2. Also which is the best, quickest, and cheapest place from which to obtain a maternity certificate?—*Nurse Norah.*

You will hardly be likely to obtain district work unless you have been trained as a nurse and have a certificate to that effect. 2. The British Lying-in Hospital, Endell Street, W.C.; the City Lying-in Hospital, City Road; the East-End Mothers' Home, &c., all offer good, cheap, and rapid course of instruction.

*Gout.*

(83) Will you kindly inform me which are considered the best places in England for taking "the waters" for gout?—*C. M. H.*

Gout is a disease of many aspects, and it would be wise to consult a medical man before undergoing a course. Bath, Buxton, Harrogate, Droitwich, and various hydropathic institutions, are frequently resorted to by sufferers from this disease.

*Address.*

(84) Would you be kind enough to give me the address of Miss Moberley, the lady who has a reading society for nurses?—*Nurse Margaret.*

24, Portland Place, W.

*South Africa.*

(85) I am very desirous of going out to South Africa to nurse, but am, unfortunately, not certificated. I have had the same amount of training as Baroness Halkett, who went out some time back, viz, 18 months, in a large London hospital, and have since held staff appointments. I have had a good deal of experience in the nursing of enteric fever, and should like, if possible, to go to Ladysmith or Kimberley to nurse. I am willing to pay my passage out, and give my services free. Do you think I should be able to get a post of any kind should I go out, or would it be any use writing to the matron at Kimberley or any other hospital to know if it would be possible to get a post? There must be work for nurses out there to do, or untrained ladies (according to the papers) would not be placed in charge of the sick and wounded.—*Canon.*

The supply of trained nurses for the sick and wounded in South Africa has far exceeded the demand. If you are trained, and prepared to pay your own expenses, you might receive reliable information where your services would be most useful—that is, if they are needed—from the Hon. Secretary, The Central British Red Cross Committee, 18, Victoria Street, S.W.

*Massage.*

(86) Will you kindly tell me: 1. The usual routine for a course of massage for the general system, and what symptoms would be noticed, should it not suit the patient. 2. Is neat's foot oil good to rub in if the patient be emaciated? 3. Which is the most instructive and latest book on massage? Having had a course of instruction some time ago I am anxious to read up all I can in connection with it.—*X. Y. Z.*

1 and 2, The patient's medical attendant is the proper person to whom to refer these questions. 3. Have you seen "The Art of Massage," by A. Creighton Hale? (price 6s., from the Scientific Press).

*Male Nurse.*

(87) Would you kindly let me know if there is an institution for training male nurses?—*Scaff.*

The Hospital for the Paralysed and Epileptic, Queen's Square, Bloomsbury, W.C., trains male nurses. Many of our male nurses are trained in the infirmary wards of the lunatic asylums.

*Indian Nursing Service.*

(88) Nurse Brown would be very glad to know where she can apply for the prospectus of the Indian Nursing Service.

A memorandum of information respecting the Indian Nursing Service is supplied by the India Office, S.W.

*Certificates.*

(89) I have had three years' experience in nursing at small hospitals, but am not certificated, so could you tell me any hospital out of London where I could get a two years' certificate? I shall be very grateful.—*E. M. E.*

See the "Nursing Profession. How and Where to Train." You would do well to train for a three years' certificate.

**Standard Books of Reference.**

"The Nursing Profession: How and Where to Train." 2s. net.
"The Nurses' Dictionary of Medical Terms." 2s.
"Burdett's Series of Nursing Text-Books." 1s. each.
"A Handbook for Nurses." (Illustrated.) 5s.
"Nursing: Its Theory and Practice." New Edition. 3s. 6d.
"Helps in Sickness and to Health." Fifteenth Thousand. 5s.

All these are published by THE SCIENTIFIC PRESS, Ltd., and may be obtained through any bookseller or direct from the publishers, 28 & 29, Southampton Street, London, W.C.

